# Born Knowing: Tentacled Snakes Innately Predict Future Prey Behavior

**DOI:** 10.1371/journal.pone.0010953

**Published:** 2010-06-16

**Authors:** Kenneth C. Catania

**Affiliations:** Department of Biological Sciences, Vanderbilt University, Nashville, Tennessee, United States of America; Georgia State University, United States of America

## Abstract

**Background:**

Aquatic tentacled snakes (*Erpeton tentaculatus*) can take advantage of their prey's escape response by startling fish with their body before striking. The feint usually startles fish toward the snake's approaching jaws. But when fish are oriented at a right angle to the jaws, the C-start escape response translates fish parallel to the snake's head. To exploit this latter response, snakes must predict the future location of the fish. Adult snakes can make this prediction. Is it learned, or are tentacled snakes born able to predict future fish behavior?

**Methods and Findings:**

Laboratory-born, naïve snakes were investigated as they struck at fish. Trials were recorded at 250 or 500 frames per second. To prevent learning, snakes were placed in a water container with a clear transparency sheet or glass bottom. The chamber was placed over a channel in a separate aquarium with fish below. Thus snakes could see and strike at fish, without contact. The snake's body feint elicited C-starts in the fish below the transparency sheet, allowing strike accuracy to be quantified in relationship to the C-starts. When fish were oriented at a right angle to the jaws, naïve snakes biased their strikes to the future location of the escaping fish's head, such that the snake's jaws and the fish's translating head usually converged. Several different types of predictive strikes were observed.

**Conclusions:**

The results show that some predators have adapted their nervous systems to directly compensate for the future behavior of prey in a sensory realm that usually requires learning. Instead of behavior selected during their lifetime, newborn tentacled snakes exhibit behavior that has been selected on a different scale—over many generations. Counter adaptations in fish are not expected, as tentacled snakes are rare predators exploiting fish responses that are usually adaptive.

## Introduction

**Figure 1:**
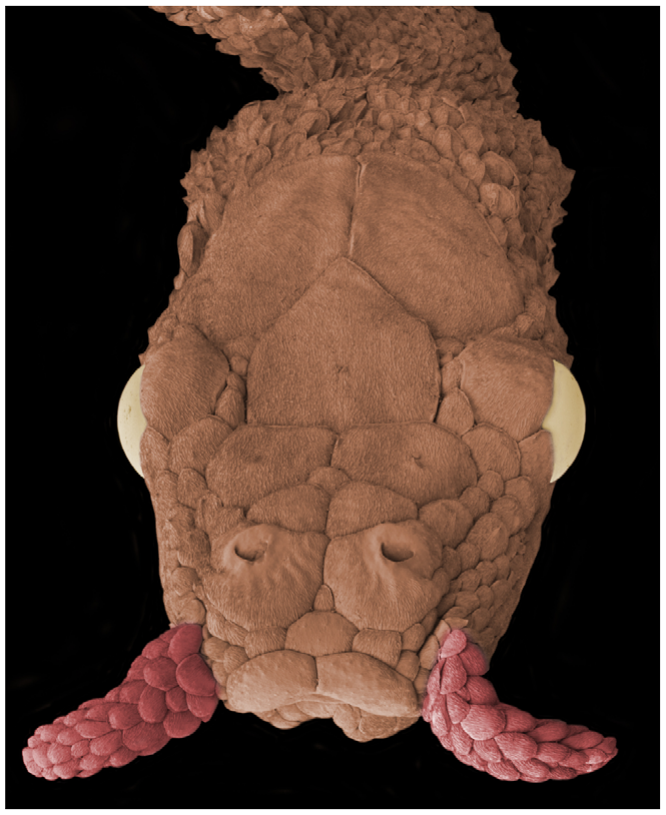
The unusual anatomy of the tentacled snake's head under the scanning electron microscope. The scaled tentacles are sensitive mechanoreceptors that respond to water movements [5]. These appendages likely aid snakes in locating fish at night, with the eyes the playing the dominant role under lighted conditions.

Aquatic tentacled snakes (*Erpeton tentaculatus*) live and breed in stagnant or slow moving water in Thailand, Cambodia, and South Vietnam [1], [2]. They are often called the “fishing snake” and this name is well earned, as they feed exclusively on fish and have unique anatomical and behavioral specializations for capturing their elusive prey [3]–[5]. Their most obvious, defining specialization is a pair of tentacles that protrude from the rostral margins of the head (Figure 1). The tentacles are sensitive mechanoreceptors that respond to nearby water movements [5]. Mechanosensory information from the tentacles projects to the optic tectum [5], a brain structure dominated by visual inputs but that also receives and integrates information from other modalities to guide attention and orientation movements [6]–[8]. Tentacle inputs activate cells primarily in deep layers of the tectum. These findings are consistent with behavioral observations that suggest vision plays the dominant role in guiding strikes at fish, with the tentacles providing additional information that becomes increasingly important as vision is degraded at night or in turbid water.

**Figure 2:**
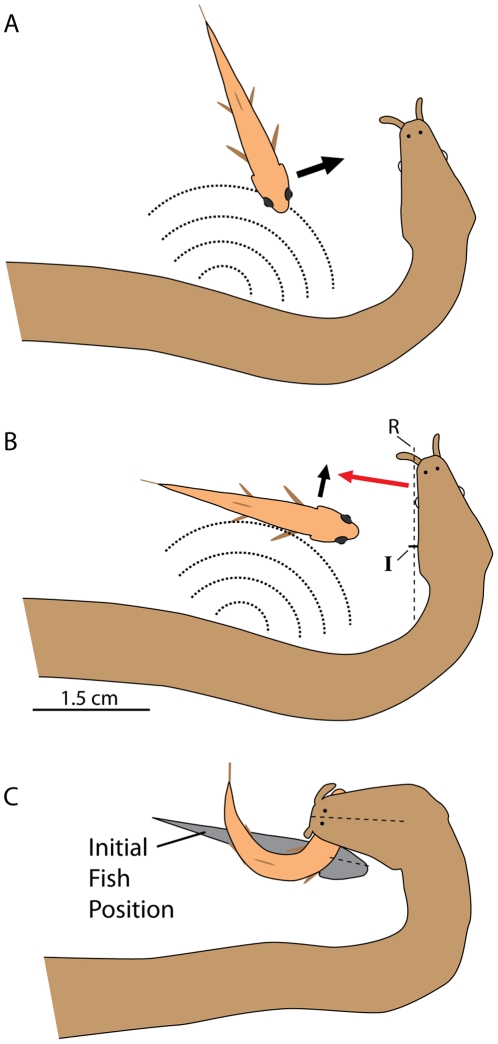
Tentacled snake hunting posture and strategy. A. When fish approach the concave area between head and body, the snake feints with its body before striking, generating a water disturbance that usually startles fish toward the striking jaws [12]. B. When fish approach the jaws at a right angle, the body feint usually startles fish away from the body. Adult tentacled snakes bias their strike to predict this future fish movement (C). The current study examines this behavior in naïve, juvenile tentacled snakes. Trials were included in the present investigations if long axis of the fish intersected line segment “R” in B, drawn from the tip of the tentacle through the long axis of the snake's jaw to the neck, and included strikes at both approaching and receding fish. The average intersection point for the long axis of fish on the snake's jaws is indicated at “I”, 11.6 mm from the tip of the head not counting the tentacle. The standard deviation for the intersection point was 5.6 mm for the 100 trails examined using a plastic barrier between snake and fish (see text and Figure 3).

In addition to the mechanoreceptive appendages, tentacled snakes have a remarkable behavior that takes advantage of the C-start escape response exhibited by fish [9]–[11]. When hunting, a snake assumes a J-shaped position with its head forming the base of the J. From this rigid posture, it waits cryptically for fish to approach and most often strikes when fish have entered the concave region between its head and body (Movie S1). Just before a strike is initiated, the snake moves its body in a “feint” that usually triggers the fish escape response away from the body and toward the head [12]. When fish are oriented parallel to the long axis of the head, the body feint provides an obvious advantage by most often startling fish in the wrong direction (from the fish perspective) - toward the snake's approaching jaws (Figure 2A). But when fish are oriented at a right angle to the jaws, the escape response translates fish parallel to the snake's head -to either the left or the right - rather than toward the strike. To exploit this latter response, the snake feints with its body and then biases its strike to the future location of the escaping fish's head (Figure 2B,C). This is remarkable, because the ballistic strikes are initiated before fish begin to move, and thus the snake must predict the future behavior of fish [12]. Is this prediction learned, or has evolution tailored the snake's nervous system to compensate for future fish movements from birth? The most likely answer is not obvious, because the body feint prior to striking is adaptive for a range of fish approach angles that do not require a predictive strike. In this investigation high-speed video analysis of snake strikes and fish escape responses reveal that naïve tentacled snakes can make an accurate prediction of future prey movement for some fish positions and orientations. In addition to these observations, prey handling time and potential predator avoidance strategies [4] are discussed for both juvenile and adult tentacled snakes.

## Results

### Predictive Strikes by Naïve Snakes

**Figure 3:**
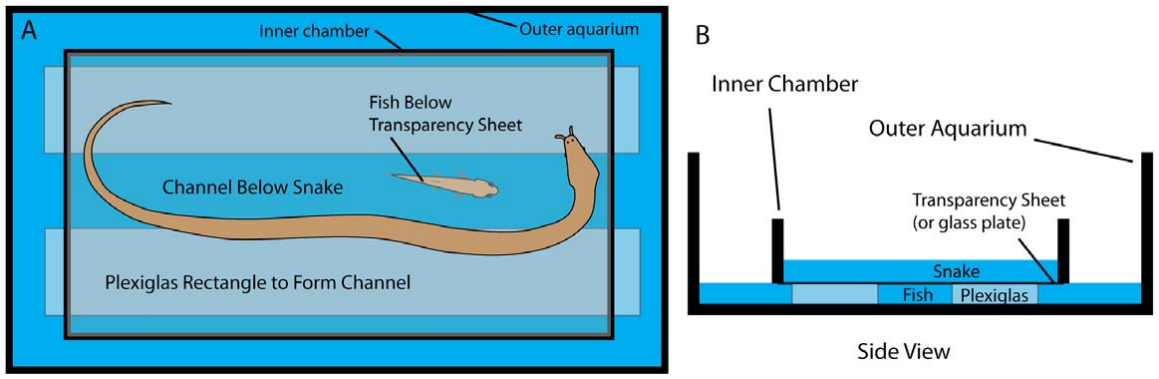
Schematic illustrating the paradigm used to prevent learning from experience. A. Top view (camera view) of the apparatus. An upper water chamber with a bottom formed from a transparent plastic barrier contained the snake and was placed into the larger, lower chamber containing the fish. The upper chamber rests on two clear plastic supports creating a channel below the snake, separated from the snake by the clear barrier. A single fish was introduced in the channel. Thus snakes could see and strike at the fish without making contact. B. Side view showing the relationship between the two water chambers.

An obvious challenge for this investigation was to prevent learning, through experience with fish C-starts, when the newborn snakes were fed fish during the course of this study. This was addressed by feeding snakes dead fish by hand, which they readily accepted. To examine strikes at live fish while preventing learning, snakes were placed in a shallow water container with a clear transparency sheet (n = 10 snakes) or glass bottom (n = 2 snakes). The chamber was placed over a channel in a separate aquarium such that a fish (a fathead minnow, *Pimephales promelas*) could swim below (Figure 3 and see methods for more details). Thus snakes could see and strike at the fish, without contact. Surprisingly, the snake's body feint elicited C-starts in the fish below the transparency sheet, allowing the accuracy of the snake's strike direction to be measured in relationship to the C-starts. C-starts were never elicited through the glass barrier, indicating that acoustic or hydrodynamic (rather than visual) components of the body feint elicit the C-starts through the plastic barrier [9], [11], [13]–[18].

**Figure 4:**
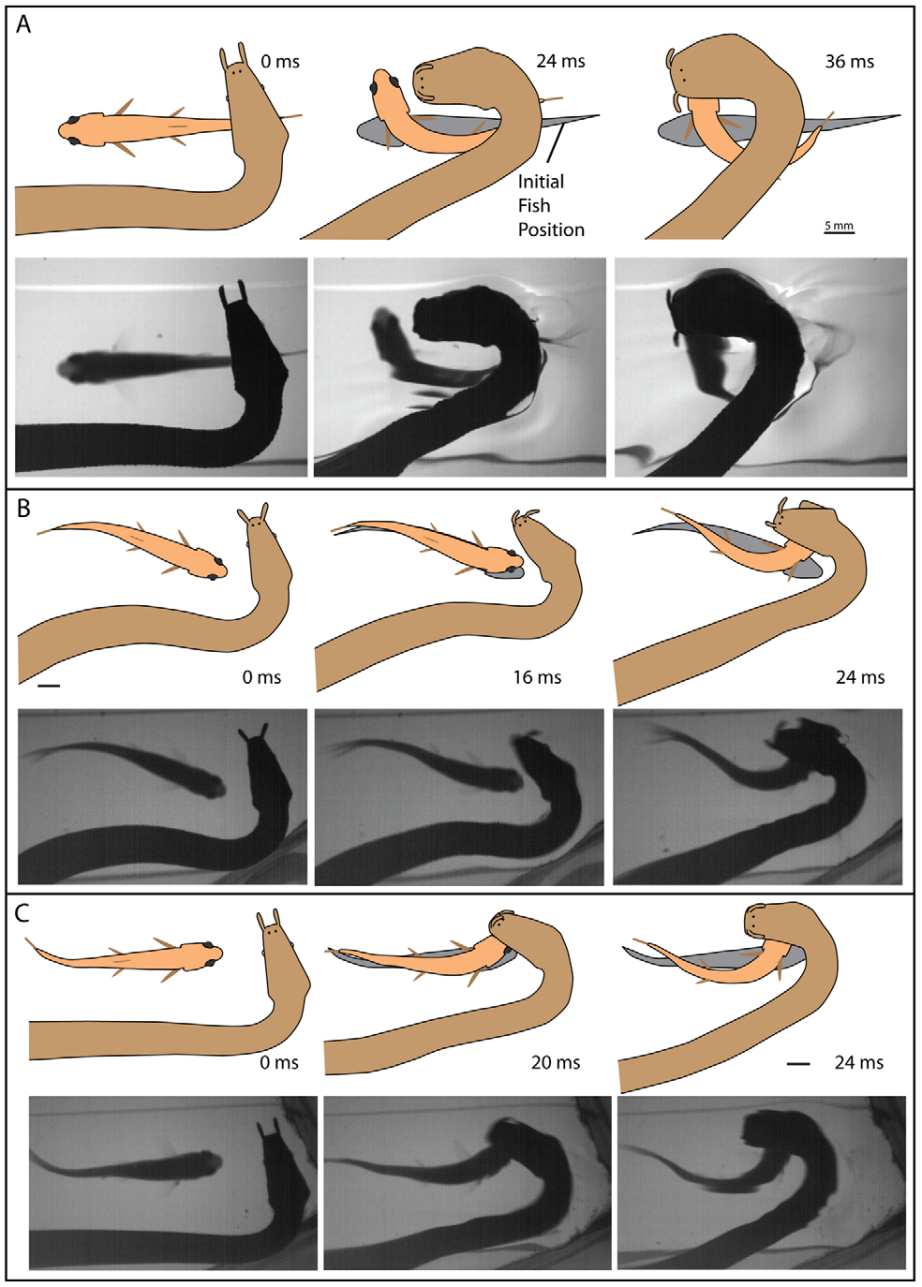
Strikes by naïve snakes at a fish below a transparent plastic barrier. A–C show three different strikes at fish. The upper panels illustrate the movements of snake and fish with the initial position of the fish marked in gray. Matching frames from high-speed video are shown in the lower panels. A small movement of the snake's body just prior to striking elicits a C-start escape response in the fish (despite the barrier) and the snake strikes toward the future position of the fish head. The barrier prevents contact between the snake and fish (see Figure 3 and Movie S2, clips 1–10). For ease of comparison, snake orientation may have been flipped to show a left-hand bend in the neck for these and other figures.

**Figure 5 pone-0010953-g005:**
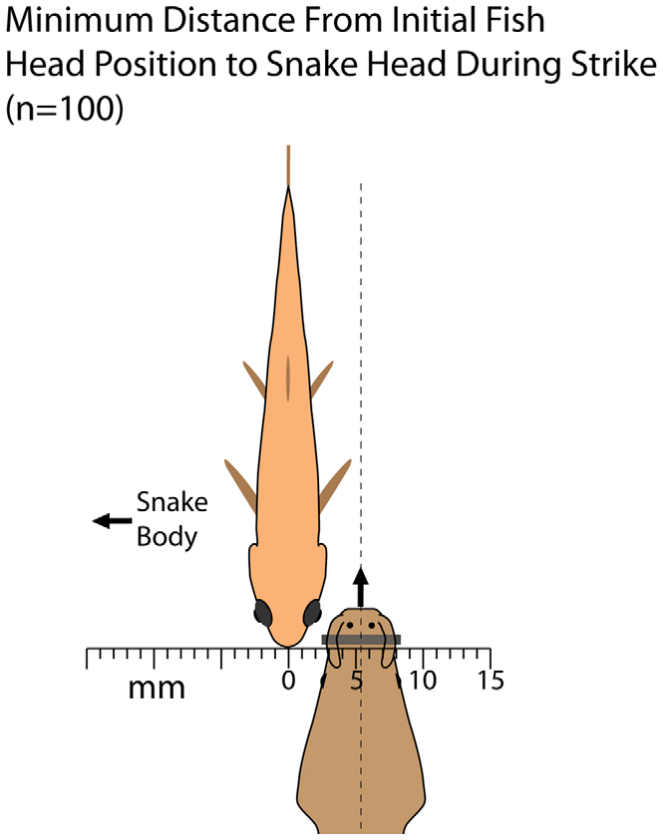
Schematic illustration of predictive strikes by tentacled snakes. The (mean) minimum distance between the midline of the snake's head and the midline of the fish head for 100 strikes (10 for each of 10 snakes) was 5.3 mm to the far side of the fish (illustrated as if the snake's body was to the left for all trials - see figure 2B,C). The standard deviation (3.0 mm) is indicated by the gray bar.

In 100 trials (10 for each of 10 snakes with fish approaching or receding at an approximate right angle to the jaw) using the plastic barrier, C-starts were elicited 88 times. Of these, 74 (84% of C-starts) were directed away from the snake's body feint. Snakes biased their strikes to the side of the fish (Figure 4), such that the snake's jaws and the fish's translating head usually converged in these trials (in 2 dimensions) as the fish escaped (see Movie S2, clips 1–10). The predictive nature of these strikes was quantified by measuring the shortest distance between the midline of the snake's head during the strike and the midline of the fish's head (Figure 2C) in its original position (at strike initiation). The average distance between the two, from the 10 snakes, was 5.3 mm (Figure 5) with a standard deviation of 3.0 mm.

**Figure 6:**
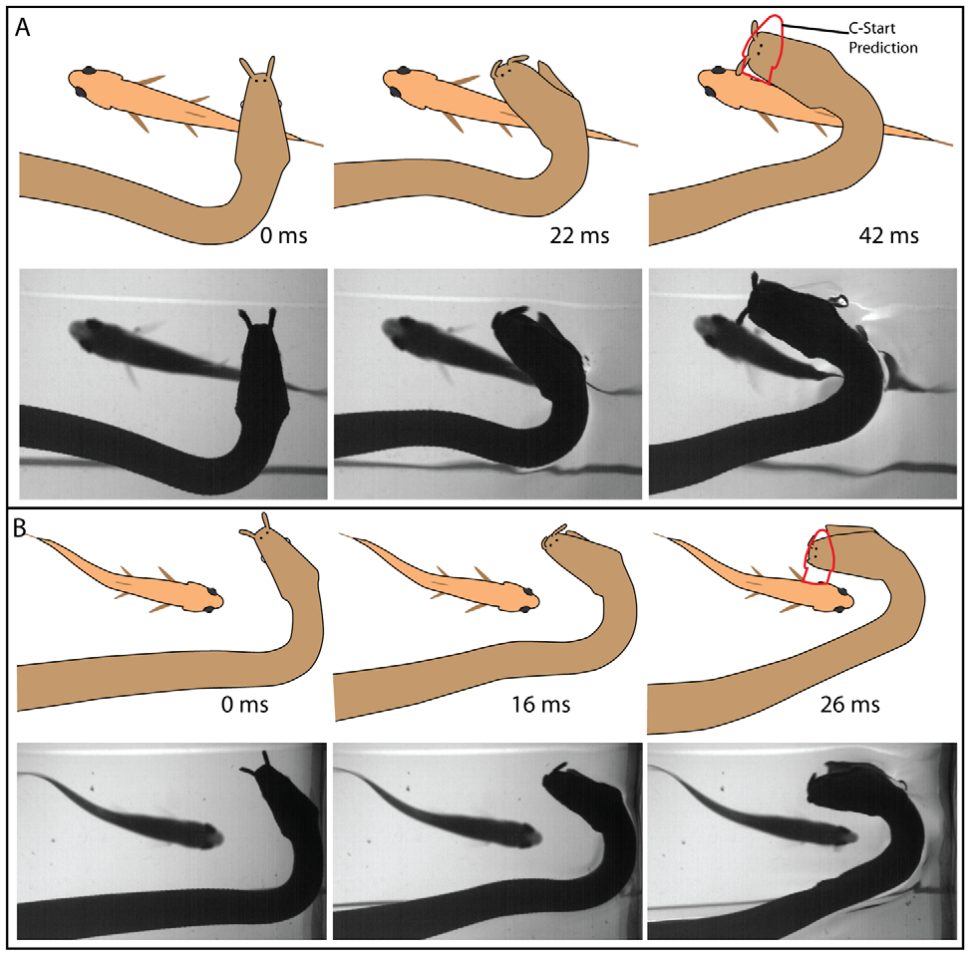
Snake strikes at fish below a glass barrier. A-B. Snakes did not elicit C-starts in fish below a glass barrier, but still biased their strikes to the far side of the fish (see text). The red outline is a projection of the estimated position of the fish head (see figure 7) during a C-start showing the presumed target of the snake.

It is important to keep in mind that snake strikes are ballistic and cannot be corrected for fish movement. There are several reasons for this conclusion that are not obvious when viewing slow-motion clips. For example, the entire strike lasts roughly 30–40 milliseconds, thus the speed and momentum of the strike were not compatible with collecting sensory information and adjusting strike direction (see Movie S1 and [12]). More direct evidence that strike aim is independent of fish movement in the present study comes from the trials using the glass barrier (Figure 6) during which no C-starts were elicited and snakes still biased their strikes to the side of fish (mean 6.0 mm, 2 snakes, 10 trails each; see Movie S2 clips 11–15).

**Figure 7:**
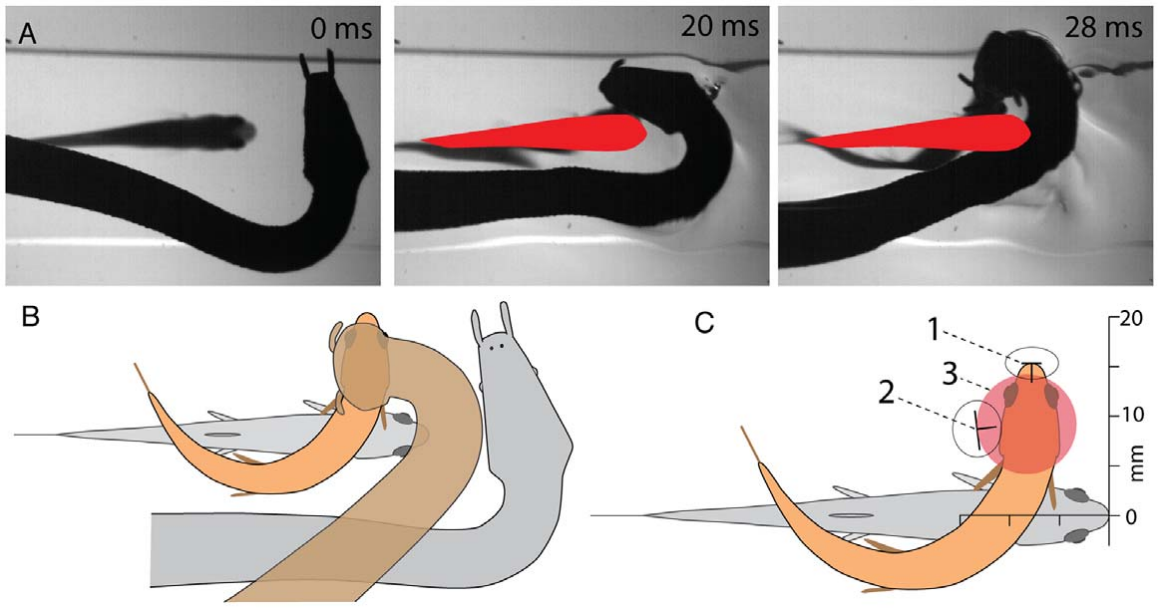
Strikes aimed down at the escaping fish. A. Selected frames from high-speed video showing a downwardly aimed strike at a fish below the plastic barrier. As the fish executed a C-start escape, the snake's strike intersected the position of the moving head (see Movie S2, clips 16–25 for examples). Red outline indicates the initial position of the fish when strike began. B. Schematic illustration of the strategy and result for downwardly aimed strikes. Note that the snake's head and jaws are poorly positioned to capture a fish that has not executed a C-start, even if aimed directly at an immobile fish – i.e. the jaws could not encircle the fish along its midline. C. Relative position of fish and snake's aim for 21 downwardly aimed strikes. The gray outline indicates the initial position of the fish, which served as a reference for the X and Y axes. Point “1” marks the mean position of the tip of the fish head after 26 milliseconds measured from ten C-starts. Point “2” marks the mean position of the top, midline of the snake's head at the time it rotated down to contact the barrier, which took a mean of 26.3 milliseconds for the 21 trials. Ellipses have radii of one standard deviation for the X and Y dimensions for points 1 and 2. The red sphere, “3” has a 1 cm diameter indicating the approximate aim of the snake.

The analysis above provided a rough estimate of the predictive nature of strikes in showing a bias to strike at the far side of the fish, in the direction of most C-starts. However during a subset of trials (n = 21) snakes aimed their strikes down at the fish, allowing an estimate of aim in X-Y coordinates relative to the fish and more precisely in time (Figure 7). The downward aim was measured from the vertically rotated head as it contacted the clear barrier, which took a mean of 26.3 milliseconds from strike onset. The position of the upper midline of the snake's head was plotted in X-Y coordinates using the long axis of the fish as the X-axis and the tip of the fish head as the origin (Figure 7C, #2). The estimated future position of the fish was plotted and averaged (Figure 7C, #1) on the same axes by measuring the tip and long axis of the fish's head 26 milliseconds after strike initiation, for 10 unobstructed C-starts (not illustrated). These data reveal an accurate predictive downward strike in naïve snakes that coincides with the average future location of the head of the C-starting fish in space and time (Figure 7C, #3) – a result clearly evident in behavior trials (see Movie S2, Clips 16–25). Examples of these downward strikes were later observed when the plastic barrier was removed and snakes captured fish for the first time (Movie S2 clips 26–27). Note that snakes bent their neck to both the left and the right when waiting to strike, but for ease of comparison in the figures and movies, the orientation of the snake is usually shown as a left-hand bend of the neck.

### Evidence for Other Predictive Strikes

The predictive strikes examined above were investigated for only a small fraction of the potential orientations of fish relative to snakes - i.e. for fish oriented at approximately right angles to the jaw and relatively close to the snake's head and body. These strikes were convenient to examine because they were common and involved primarily only 2 dimensions of movement that can be readily measured from video recordings. However in the course of filming both naïve and adult tentacled snakes, two other forms of apparently predictive strikes were observed. These are briefly described below, first for observations from experienced adult snakes, and then evidence for these strikes is described for naïve juveniles that had never experienced fish escape responses. These observations suggest that tentacled snakes are born with a variety of strike strategies that might be used depending on variations in fish orientation relative to the head.

#### Twisted Strikes

**Figure 8:**
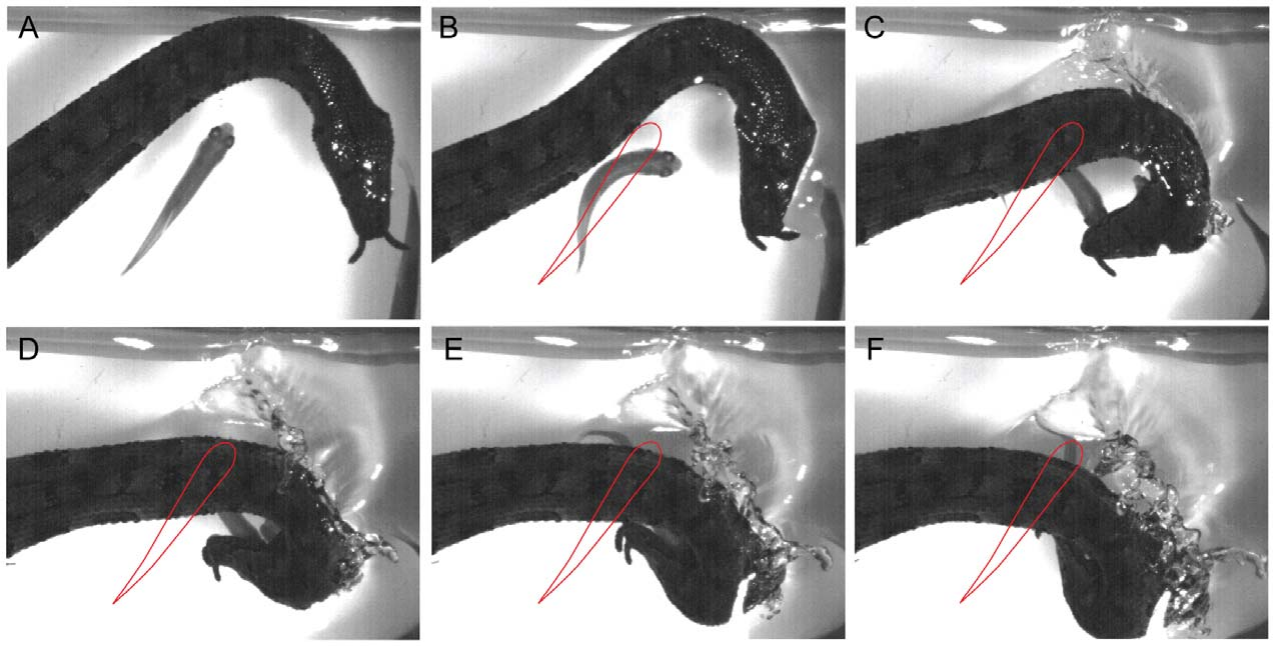
A twisted strike. Selected frames of an adult tentacled snake strike during which the head is curled around and under the body to meet an escaping fish head-on. The red outline marks the original position of the fish. These strikes were observed in the naïve tentacled snakes striking at fish below a barrier (Movie S3, clip 8) and striking at fish for the first time (Movie S3, clip 9).

One class of unique strikes, first observed in adults, was evident when fish were located below the plane of the snake's head and body and had moved relatively close to the area of the snake that generates the body feint. In this case, snakes sometimes captured fish by eliciting a C-start, and then twisting their head around and under their own body to meet the escaping fish head-on (Figure 8 and Movie S3 clips 1–7 for adults). As in the strikes previously described, these were clearly predictive strikes because they began before the fish moved yet later intercepted the moving head of the fish. As was the case for other strikes, this conclusion was supported by examples when fish did not C-start and snakes still struck to the predicted location of the escaping fish head. The latter was observed for both adult snakes striking at unobstructed fish (Movie S3 clip 7) and naïve snakes striking at a fish below a glass barrier, during which C-starts were never elicited (Movie S3 clip 8). When naïve snakes were later presented with live fish and no barrier, these strikes were employed to capture fish - for one snake on the first strike and capture (Movie S3 clip 9). For snakes to succeed with these strikes, the fish must essentially insert its head into the wedge shaped area created by the rapidly moving upper and lower jaws of the snake. Thus as was the case for the strikes aimed downward, these strikes reveal an accurate prediction of the future location of the escaping fish head.

#### Range Extending Strikes

On a number of occasions, adult and juvenile tentacled snakes struck at fish that were near the limit, or completely outside, of their apparent strike range. However the snake's body feint and subsequent fish C-start shortened the distance between snake and fish making capture more likely. Although the snake often missed by striking either above of below the target, the fish had moved to a position that made capture feasible and occasionally successful (Movie S3, clips 10–13). These strikes were also observed in the naïve juveniles (Movie S3, clips 14–15).

### Handling Time and Strike Strategy

**Figure 9:**
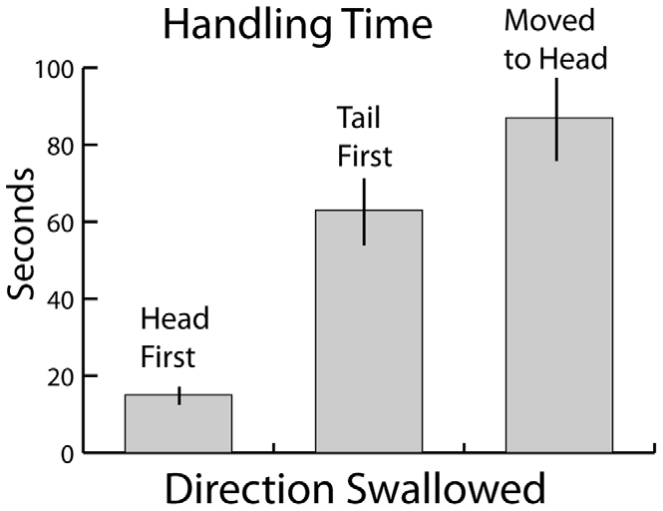
Handling time for swallowing fish from different directions. Each condition included 10 trials, bars are standard deviation. Handling time was defined as the time from strike initiation until the fish completely disappeared from view from the dorsal perspective. The far right column refers to fish that were grasped tail-first, but then manipulated into a head-first position for swallowing.

As described above, tentacled snakes usually aim for the head of fish and are often successful at striking their target. Previous investigators have noted an exceptionally short handling time corresponding to this strategy in tentacled snakes [2]. To investigate the effect of strike location on handling time juvenile snakes where fed fish by hand either head first or tail first. For these measures, handling time was defined as the time from strike onset until the fish had disappeared completely from view from the dorsal perspective. Three different behaviors were observed and timed for ten trials each: 1) head first capture and swallow, 2) tail first capture and swallow, and 3) tail first capture followed by manipulation of the fish for a head first swallow (Figure 9). As might be expected, the head-first capture and swallow corresponded to by far the shortest handling time with a mean of 15 seconds. Tail-first capture and swallowing was 4 times longer (mean 63 seconds) whereas manipulating the fish from a tail-first capture for a head-first swallow took a mean of 87 seconds. The latter examples are conservative estimates of tail-first swallowing time, as 1 trial was excluded during which the snake was unable to swallow the fish past the gills within 30 minutes.

## Discussion

The main goal of the present investigation was to investigate the hypothesis that tentacled snakes are born with an innate ability to startle fish and then predict and aim for the future location of an escaping fish's head. This possibility followed from a previous investigation of adult tentacled snakes that exhibit this ability [12]. The evidence collected here from laboratory-born, naïve snakes shows that this ability is, at least in part, innate. This was shown for 3 different striking strategies: 1) snakes biasing strikes to pass to the side of the initial fish position toward the direction of a C-start escape (Figures 4–6 and Movie S2. clips 1–10), 2) snakes striking down at the future location of the top of the fish's head (Figure 7 and Movie S2, clips 16–25), and 3) snakes curling their head around and down during the strike to meet the escaping fish head-on (Figure 8 and Movie S3, clips 1–9). The predictive nature of strikes during which fish were startled into range remains more tentative.

### Predictive Strikes

Most of the predictive strikes exhibited by snakes in this investigation were elicited by fish located relatively close to the body of the snake, such that a line drawn through the long axis of the fish intersected the head of the snake proximal to the eye (Figure 2B). When the long axis of the fish intersected the snake's head distal to the eye (e.g., Movie S2, clips 20 and 23), the strike seemed less likely to be predictive (i.e. was more often closer to or overlapping the original position of the fish at strike initiation). This follows naturally from several aspects of the snake's hunting position. For example, it is difficult for snakes to strike around to the far side of more distantly located fish. At the same time, the snake's body feint is less likely to elicit a C-start for fish more distant from the snake's body, presumably making a predictive strike less appropriate. Finally, when fish are very close to the snake's body and head, it may be impossible for the snake to strike directly at their position, as the angle is too acute. In such cases the only strategy for capture may be to elicit an escape response that propels the fish to within range. In contrast to the situation with more distant fish, in the latter case fish are located particularly close to the area of the snake's body that generates the feint stimulus, and are therefore most likely to respond with a C-start and hence “fall for” the predictive strike.

The most common predictive strikes were those simply aimed to the side of the fish's head–in the most likely direction of a future C-start. But even more compelling for their predictive accuracy were the downwardly aimed strikes at the future location of the fish head (Figure 7), and the strikes during which snakes twisted their head around and under to meet escaping fish head-on (Figure 8). Rather than simply sweeping through the likely axis of the escaping fish's path, these latter cases required more precision in space and time such that the wedge shaped opening of the snake's jaws moving in one plane, met the fish's moving head in another plane. In both of these cases, the strikes would be unlikely to succeed in the absence of a C-start even if they had been aimed directly at an immobile fish, because the tips of the jaws would strike the long axis of the fish preventing encirclement (see Figure 7B).

It should be emphasized that the predictive nature of the strikes described here is qualitatively different from predictions of prey (or object) position based on initial movements and trajectories [19], [20]. Predicting the future position of a moving object is common, and equivalent to a batter striking a baseball based on brief visual information about its initial trajectory. Extending this analogy to tentacled snakes–it would be as if the batter could estimate the position of the ball in time and space without ever seeing the pitch. Moreover, the present study of naïve snakes shows they can do this without ever seeing any pitches. Of course the situation is simplified for the tentacled snakes because they elicit a specific pitch (the C-start), providing the basis for their precisely timed strike. Nevertheless, the fact that “knowledge” of C-starts is innate and therefore inherited by tentacled snakes is remarkable, and seems to be unparalleled.

### The Cue Eliciting C-Starts

The use of a thin transparency sheet barrier between the snake and the fish was to prevent the snake from learning through experience capturing fish. Thus snakes struck at the fish but were never able to make contact during the trials. It was fortuitous that the fish C-started in response to the body feint despite the barrier, as this allowed a comparison of movements between the snake and fish. Replacement of the plastic barrier with a glass plate eliminated all C-starts in response to snake strikes, despite the close proximity of snakes to the fish. This strongly suggests that C-starts were elicited by hydrodynamic or acoustic components of the body feint, transduced by the ears or lateral line mechanoreceptors (or both), rather than by visual stimuli. The fish behavior is consistent with the short latency of the C-start response to acoustic stimuli compared to vision [21]–[24]. This conclusion was previously suggested based on hydrophone recordings of striking snakes [12].

It should be noted that a single fish was used for all of the 100 trials analyzed for the plastic barrier and for the 20 glass plate barrier conditions in which C-starts were eliminated. This was done to facilitate measurements, as no correction was needed to normalize data relative to variations in fish size across trials. It is interesting that the fish never habituated to strikes. Presumably this is consistent with the critical role of the C-start in the context of a predatory strike - i.e. it would not be adaptive to habituate to a striking predator. However it was subsequently found that not all fish were as responsive to snake strikes through the transparency sheet barrier. Although this investigation was focused on the behavior of snakes, the behavior of fish is often the focus of such studies. It therefore seemed worth exploring and potentially improving the barrier condition for eliciting C-starts from a separated predator. It was found that replacing the transparency sheet barrier with a tightly stretched polyvinyl film (i.e. Saran Wrap or Stretch-Tite) that was thinner and more flexible provided a more efficient barrier for allowing transmission of snake-generated stimuli that elicit C-starts. Using this configuration, additional trials were collected from a number fish and it was confirmed that visual cues alone (snakes striking with a glass plate barrier in place) never elicited C-starts whereas the body feint was efficient for a range of different fathead minnows (*Pimephales promelas*). These experiments were then further extended to include wild-caught *Gambusia affinis* and the same basic result was observed in the course of 10 trials (see Movie S3, clips 16–18 for the 3 types of predictive strikes by juvenile snakes through a polyvinyl film with wild caught *Gambusia* as prey).

The widespread occurrence of C-starts in fish [25] provides a context for the evolution of the exploitative behavior of tentacled snakes. Many different predators feed on fish making tentacled snakes the classic “rare enemy” [26]–[30] able to exploit the cues that usually elicit an adaptive behavior. If tentacled snakes are rarely encountered compared to other predators, as seems likely, then counter adaptations in fish may never evolve, as a rapid turn away from water disturbances is most often the best response.

### Snake Strikes and Learning

Although the results of the present investigation indicate that tentacled snakes are born able to predict the future movement of their prey, they still learn from experience. At least one facet of learning was clearly observed in the present investigation - snakes stopped striking at the fish below a barrier over time. Most snakes were hesitant to strike after about 10 trials, and two snakes were not included in the analysis because they struck less than 10 times in total (at fish below a barrier). Snakes continued to strike at fish in other positions (e.g. dead fish fed by hand, and later at live fish in other positions), thus the feedback of striking a barrier was learned quickly. It is possible they also learn to adjust their aim over time based on feedback from fish captures, despite the predisposition to account for the C-start from birth.

There may also be learned or innate changes in strike strategy with growth and development. One potential difference observed between large snakes and small snakes was the magnitude of the body feint. The largest female snake (77 cm total length) seemed to exhibit an exaggerated body feint when startling fish (Movie S3, clips 19–20). Possibly it takes a larger body feint to elicit C-starts in larger fish. In any case, the relationship between neuromuscular development, innate modifications of behavior to accommodate body size, and learning from experience, would be difficult to determine. It seems likely that all of these factors could play a role in modifying strikes over time.

### Advantage of Strike Strategies

The ability to strike the head of an escaping fish from the time of birth may provide a critical advantage to neonates that are particularly vulnerable to predation or starvation. The most obvious advantage of striking the head is an increased likelihood of successful capture. The head is the thickest part of the fish whereas the body and thin, mobile tail may present more elusive targets. But there are other reasons to aim for the head. Some fish erect spines on their dorsal fins that could damage the snake's mouth during a high-speed strike [31], [32]. In addition, even after a fish is successfully grasped, there is a substantial difference in handling time depending on the capture point (Figure 9). Fish that were swallowed head-first had by far the shortest handling time, whereas fish that were swallowed tail-first had a longer handling time and may get lodged in the snake's mouth at the gills, as occurred for at least one trial in the present study. In addition, fish with spines may be impossible to swallow tail first, requiring even longer handling times as they are manipulated into a new position (Figure 9) or eventually released. Handling time, in turn, may be more important than simply improving foraging efficiency [33]–[38]. Previous investigators have noted a tail-wiggling behavior in tentacled snakes when swallowing prey [4]. This has been interpreted as a defense mechanism that distracts the snake's potential predators [4]. Presumably a predator that strikes the tip of the snake's tail is unlikely to succeed in capturing the snake. The same tail-wiggling behaviors were observed in the present investigation, sometimes in response to a shadow passing over the snake's position. This anti-predator interpretation seems likely, and suggests that tentacled snakes are in danger of being seen and attacked when handling fish. Thus minimizing handling time would be adaptive not only for foraging efficiency, but also for maximizing camouflage and thus survival. Finally, minimizing handling time might also reduce the likelihood that other, nearby fish would observe the snake and avoid its location.

These various advantages have presumably been significant over the long evolutionary history of tentacled snakes feeding on fish, allowing selection to act on innate behaviors. This level of selection of behavior can be contrasted with learning, which selects behavior during the animal's lifetime. The result is a predator born with future prey movements in mind.

## Methods

### Husbandry

Several adult tentacled snakes were housed together in a 60-gallon (227 liter) glass aquarium with water between pH 6.5 and 7, gravel, plastic plants, and artificial branches. Two 50-watt submersible heaters were used to maintain water temperature between 25 and 28°C. The cage was secured using a screen-top and clamps, and topped with a 20-watt fluorescent fixture on a 12/12 h light/dark cycle. Snakes were given 8 hours of *ad libitum* access to goldfish (*Carassius auratus*) three times each week. Litters from two of the snakes were born on the 17th and 19th of September, 2009 and included 11 and 7 surviving young respectively. Neonatal snakes were removed from the tank immediately after birth and were group-housed in 5 and 10-gallon glass tanks that were similarly outfitted with the same water parameters and lighting conditions. Once weekly juvenile snakes were fed 1–2 dead fathead minnows *(Pimephales promelas)* by hand during the course of the study.

### Behavior Trials

For behavior trials, juvenile snakes were placed in a 10×18 cm Plexiglas enclosure with a transparency sheet (3M PP2200), polyvinyl film (Stretch-tite food wrap), or glass (2.5 mm thick) bottom filled with 1–2 cm of water. This was placed onto 2, 9 mm thick 6×25 cm Plexiglas spacers in a shallow aquarium that formed a channel below the clear barrier (Figure 3, outer aquarium not to scale). For the transparency sheet and glass plate trials analyzed in the study, a single fathead minnow (*Pimephales promelas*) was used. The minnow was introduced into the channel below the snake to elicit strikes. Trials were filmed with a MotionPro HS-3 camera (Redlake) at 250 or 500 frames per second and video was transferred to a MacPro laptop using MotionProX software. Trials with the polyvinyl film barrier used both fathead minnows and wild-caught Gambusia (*Gambusia affinis*). Note that snakes bent their neck to both the left and the right when waiting to strike, but for ease of comparison in the figures and movies, the orientation of the snake is usually shown as a left-hand bend of the neck. All procedures were approved by the Vanderbilt Institutional Animal Care and Use Committee and are in accordance with the National Institutes of Health guidelines for the care and use of animals in research (Animal Welfare Assurance Number A-3227-01).

### Measurements of Snake and Fish Positions

To illustrate strike aim and initial fish position in the figures and movies, a mask was made over the initial fish position (Movie S2), in Adobe Photoshop, in the frame corresponding to the first visible snake movement. This mask was then transferred, in the same coordinates, to subsequent frames to facilitate comparison of strike aim to initial fish position. Distances from the fish initial head midline to snake's head midline were measured directly from selected frames. The frame containing strike onset and the frame with the closest pass of the fish initial position were exported to Adobe Illustrator. The midline of the snake and fish head where marked with a vector line, and distances were measured using filmed scale bars. For transparency sheet (10 trials each for 10 snakes) and glass barrier conditions (10 trials each for 2 snakes), trials were included if the long axis of the fish intersected line segment “R” in figure 2B, drawn from the tip of the tentacle through the long axis of the snake's jaw to the neck, and included strikes at both approaching and receding fish. However trials of downwardly aimed strikes (Figure 7) included only approaching fish, as too few receding fish trials were obtained for separate analysis. For position measurements in these trials, the position and angle of the top of the snake's head was marked on a transparency sheet relative to the original fish position, and the image was scanned and placed into Adobe Illustrator, scaled and aligned to the gray schematic in figure 7C, and the grid function - scaled to millimeters - was used to determine X-Y coordinates. The downwardly aimed strikes were a subset of the 100 trials described above.

## Supporting Information

Movie S1 Examples of juvenile tentacled snakes striking at fish in real time.
(0.88 MB MOV)

Movie S2 Slow motion movie clips of naïve tentacled snakes striking. The gray mask provides a reference to the position of the fish when strikes were initiated. Clips 1–10 show snakes striking at a fish below a transparency sheet barrier. The fish usually responded to the snake's body feint with a C-start despite the barrier. Snakes aimed for the future location of the moving fish head. Clips 11–15 show snakes striking at a fish below a glass barrier. In this condition, the fish never executed an escape response, suggesting visual cues from the snake played little role in the escape response. Clips 16–25 show snakes striking downward at fish below the transparency sheet barrier. Clips 26–27 show some of the first strikes by a snake at fish without the barrier in place, illustrating the downward strike and capture.
(3.15 MB MOV)

Movie S3 Slow motion movie clips of both experienced adult and naïve tentacled snakes striking. The gray mask provides a reference to the position of the fish when strikes were initiated. Clips 1–7 show adult snakes twisting their head around and under their body when striking at fish. Clips 8 and 9 show the same strategy employed by a naïve tentacled snake striking at a fish below and glass barrier (8), and striking at the first fish introduced without a barrier in place (9). Clips 10–13 show adult snakes startling fish closer to their jaws during the strike in a manner that may extend their range. Clips 14–15 show naïve juvenile snakes employing a similar strategy striking at fish below a plastic barrier. Clips 16–18 show juvenile snakes striking and wild-caught *Gambusia* with a polyvinyl barrier in place. Clips 19 and 20 show a large adult tentacled snake striking with no barrier in place and exhibiting a more distinctive body feint to startle the fish.
(6.38 MB MOV)
